# Haploidentical stem cell transplantation with posttransplant cyclophosphamide in children with Wiskott–Aldrich syndrome: a case report

**DOI:** 10.3389/fimmu.2025.1495666

**Published:** 2025-02-04

**Authors:** Le Nguyen Ngoc Quynh, Binh Nguyen Thanh, Lien Luong Thi, Thuy Nguyen Thi Dieu, Duong Dang Anh, Pamela P. Lee, Tung Cao Viet, Dien Tran Minh

**Affiliations:** ^1^ Stem Cells Center, Vietnam National Children’s Hospital, Hanoi, Vietnam; ^2^ Pathophysiology and Immunology Department, Hanoi Medical University, Hanoi, Vietnam; ^3^ Pediatric Department, Hanoi Medical University Hospital, Hanoi, Vietnam; ^4^ Pediatric Department, Hanoi Medical University, Hanoi, Vietnam; ^5^ Surgical Intensive Care Unit, Vietnam National Children’s Hospital, Hanoi, Vietnam; ^6^ Department of Paediatrics and Adolescent Medicine, School of Clinical Medicine, Li Ka Shing Faculty of Medicine, The University of Hong Kong, Hong Kong, Hong Kong SAR, China; ^7^ Children Heart Center, National Children’s Hospital, Hanoi, Vietnam

**Keywords:** Wiskott–Aldrich syndrome, thrombocytopenia, inborn error immunity, hematopoietic stem cell transplantation, posttransplant cyclophosphamide

## Abstract

Wiskott–Aldrich syndrome (WAS) is a condition characterized by a low platelet count, eczema, and a weakened immune system. Hematopoietic stem cell transplantation (HSCT) is the only curative treatment option. Haploidentical HSCT with posttransplant cyclophosphamide (PTCy) is an emerging approach for children with noncancerous conditions. This case describes a WAS patient who was early diagnosed and successfully treated with haploidentical HSCT. A 3-month-old boy presented with widespread eczema, a low platelet count, and severe infections in infancy. The diagnosis of WAS was quickly confirmed by genetic test. He received immunoglobulin replacement therapy and antimicrobial prophylaxis and underwent HSCT at 4 years 3 months of age. After failed unrelated cord blood HSCT, second rescue haploidentical HSCT had been performed using the patient’s mother as the donor, with stem cells collected from peripheral blood. The conditioning regimen included anti-thymocyte globulin, melphalan, and fludarabine. The stem cell dose was 2.63 × 106 CD34+ cells/kg. GVHD prevention included PTCy, mycophenolat mofetil, and tacrolimus. The patient had no significant complications after the transplant. Neutrophil and platelet engraftment occurred promptly. At 32 months post-HSCT, the patient had complete hematological and immune reconstitution, with full donor chimerism and no GVHD. In conclusion, the PTCy approach to haploidentical HSCT was a safe and effective treatment for this WAS patient.

## Introduction

Wiskott–Aldrich syndrome (WAS) is an X-linked genetic disorder caused by mutations in the gene encoding the Wiskott–Aldrich syndrome protein (WASp) (1). WAS is characterized by eczema, a low platelet count, increased susceptibility to infections, autoimmunity, and/or cancer. The severity of the condition can vary widely due to different WAS gene mutations, ranging from a severe “classic” form to a milder presentation (1). Early diagnosis is essential for the management and treatment of WAS, which includes antimicrobial prophylaxis, immunoglobulin replacement therapy, and hematopoietic stem cell transplantation (1, 2).

Allogeneic hematopoietic stem cell transplantation (HSCT) is a curative treatment for WAS (2). Without HSCT, patients often develop autoimmune/inflammatory complications and have an increased risk of blood cancers, with a life expectancy of only 15 years (3). HSCT from an HLA (human leukocyte antigen)-identical sibling donor has a 5-year survival rate of greater than 90% (4). The results have also improved for matched unrelated donor HSCT, with recent studies showing over 80% survival (2, 4). However, some patients lack a suitable matched family or unrelated donor. Haploidentical HSCT from a family member who is only half-matched is another option for patients with Wiskott–Aldrich syndrome (WAS) (5). However, this approach carries a greater risk of complications such as graft-versus-host disease (GVHD) and graft failure, leading to increased transplant-related morbidity and mortality (5). To mitigate these risks with haploidentical HSCT, one strategy which has developed over the last decade is nonmanipulated haploidentical HSCT combined with posttransplant cyclophosphamide (PTCy) as GVHD prophylaxis (6–8). Cyclophosphamide administered within 72 hours of the transplant is selectively toxic to proliferating lymphocytes. It helps deplete alloreactive T cells from both the donor and recipient, facilitating engraftment and preventing GVHD (8). This PTCy-based haploidentical HSCT protocol has been increasingly used to treat adult patients with malignant diseases (7–9). In the nonmalignant disease setting, it has been applied to conditions such as sickle cell disease, thalassemia, severe aplastic anemia, and certain inborn errors of immunity (8–10).

The current case describes the successful use of haploidentical HSCT with PTCy in a 42-month-old patient diagnosed with Wiskott–Aldrich syndrome.

## Clinical case

We report the case of a male child born to nonconsanguineous parents. He was the third son of his parents. The patient had generalized itchy eczematous rashes and bloody diarrhea in the first month of age, which was interpreted as allergic proctocolitis. These disorders are refractory to a diet excluding cow milk. At 3 months of age, the boy was assessed at the National Children’s Hospital in Hanoi city, Vietnam. Physical examination revealed generalized pruritic eczematous rashes all over the body, thin and dull hair, and the presence of petechiae predominantly on the lower extremities and torso. He has been underweighted (weight-for-age below the 1^st^ percentile and height-for-age under the 2^nd^ percentile). He also had 2 episodes of otitis media and pneumonia. A full blood count revealed hemoglobin of 107 g/L, white blood cell counts of 6940/mm^3^, and microthrombocytopenia with a platelet count of 41000/mm^3^. Owing to physical examination and microthrombocytopenia, a diagnosis of inborn error immunity has been suggested. Peripheral blood examination revealed the following: IgG, 6.03 g/l (normal range: 2.32-14.11 g/L); IgA, 0.3 g/l (normal range: 0-0.83 g/L); IgM, 1.86 g/l (normal range: 0-1.45 g/L); and IgE, 13.43 IU/ml (normal range: 0-230 IU/mL). Flow cytometry revealed the following absolute lymphocyte counts: CD3+: 1729/mm^3^ (normal range: 1400-3700 cells/mm3), CD4+: 718/mm^3^ (normal range: 700-2200/mm^3^), CD8+: 639/mm^3^ (normal range: 490-1300/mm^3^), CD19+: 392/mm^3^ (normal range: 700-1100 cells/mm^3^), and CD56+: 1151/mm^3^ (normal range: 300-600 cells/mm^3^). Sanger sequencing analysis of 12 exons of WAS gene revealed missense mutation Y107C in the WASP-interacting protein (WIP) binding drosophila enabled/vasodilator-stimulated phosphoprotein homology 1 (EVH1) domain of WAS gene, confirming the diagnosis of Wiskott–Aldrich syndrome. WAS protein expression in peripheral blood cells is unavailable in Vietnam. The patient’s mother was found to be heterozygous for the mutation. As soon as the diagnosis of WAS had been made, treatment was initiated with monthly intravenous immunoglobulin (IVIG) at a dose of 500 mg/kg and the prophylactic antibiotic cotrimoxazole for *Pneumocystis jirovecii*. However, the patient had an unfavorable evolution that included chronic diarrhea and multiple pneumonias with isolation of *Streptococcus pneumoniae*, *rhinovirus* and *respiratory syncytial virus* (RSV). Additionally, he suffered from recurrent skin infections and reactivation of cytomegalovirus (CMV) with CMV retinitis while receiving treatment with IV ganciclovir. Consent for HSCT was obtained from the patient’s parents, however neither matched related donors nor unrelated bone marrow donors could be found. He was closely monitored and treated with IVIG 600-800mg/kg every 3-4 weeks, prophylactic antibiotics, and ganciclovir if needed while waiting for an appropriate donor.

At the age of 42 months, a 5/6 HLA-mismatched, blood group-matched unrelated cord blood was chosen for HSCT. The cryopreserved stem cell dose was 3.27 × 10^5^ CD34+/kg. The myeloablative conditioning regimen consisted of intravenous busulfan (1.2 mg/kg every 6 hours for 4 days), intravenous fludarabine (40 mg/m^2^/day for 4 days) and intravenous Fresenius antithymocyte globulin (ATG) (15 mg/kg/day) for 4 days. Graft-versus-host disease prophylaxis consisted of cyclosporin A and mycophenolat mofetil (MMF). Unfortunately, there was pancytopenia with no evidence of hematological recovery of donor cells by day +42 after transplant, chimerism result was 0%, and the patient was diagnosed with primary graft failure. He also suffered from CMV and Epstein–Barr virus (EBV) reactivation posttransplant.

The second rescue transplant was performed 2 months after the 1^st^ transplant. The patient was noted to have DSA (donor specific antibodies) to HLA-DR4 (detection by Luminex single-antigen with One Lambda Labscreen) of his father with an MFI (mean fluorescence intensity) of 11,783. As a result, a potential donor was his mother, who was a carrier and had the same blood group B+ and CMV (-) ve (patient B+/CMV+ve). Donor stem cells were mobilized via granulocyte colony-stimulating factor, and peripheral blood stem cells were harvested. The modified conditioning regimen for haploidentical HSCT was performed according to the European Society for Blood and Marrow Transplantation/European Society for Immunodeficiencies guidelines for HSCT for primary immunodeficiency (11), using intravenous Fresenius anti-thymocyte globulin (ATG) 15 mg/kg/day for 2 days, intravenous melphalan 70 mg/m^2^ every 12 hours for 1 day, and intravenous fludarabine 30 mg/m^2^/day for 5 days. A dose of rituximab 375 mg/m2 was added on days -13 and -5, and prophylactic foscarnet was given through the transplant to manage the EBV and CMV infections. On November 22^nd^, 2021 (day 0), the patient received a stem cell dose of 5 × 10^8 total nucleated cells/kg (CD34+: 2,63 × 10^6/kg).

**Figure 1:**
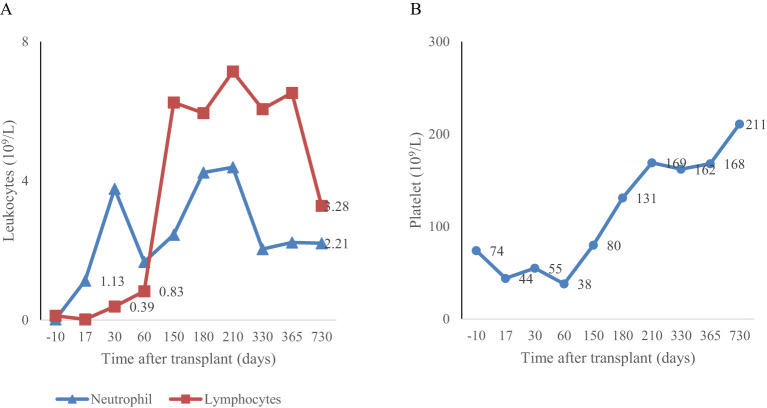
Myeloid engraftment occurred on day 17 **(A)** and platelet engraftment occurred on day 43 **(B)** after the second transplantation.

**Figure 2:**
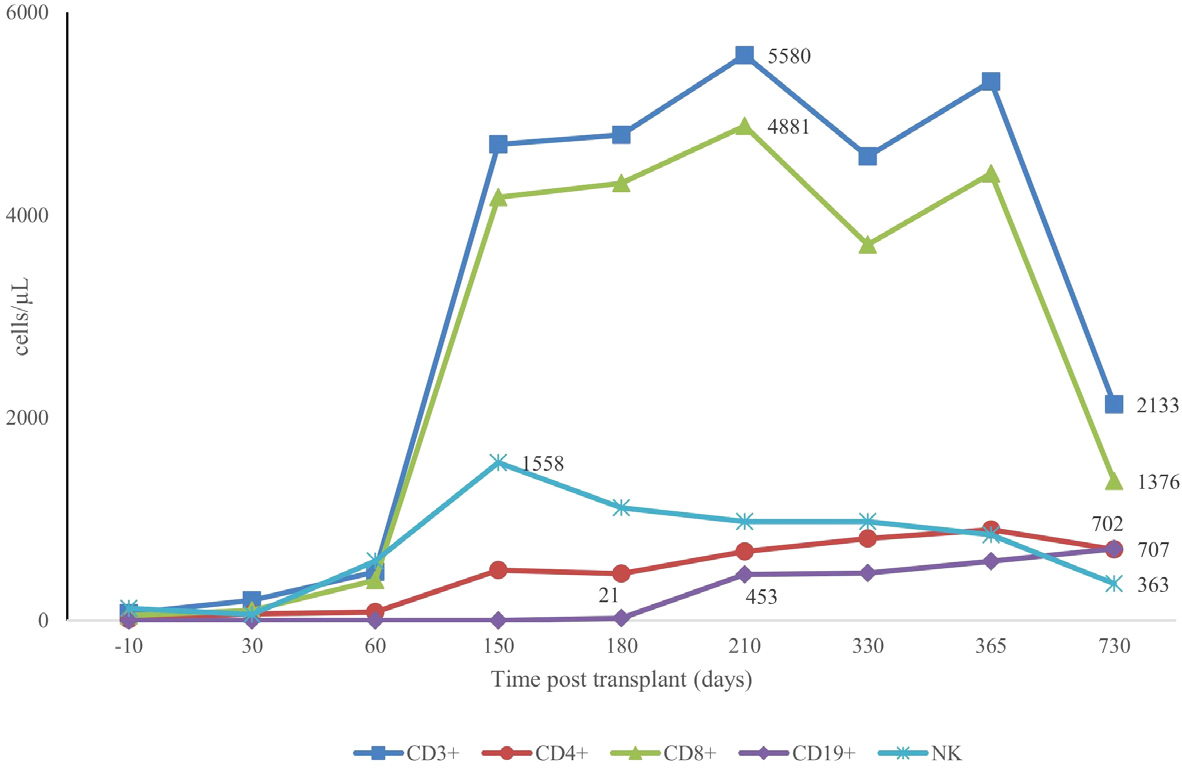
Patterns of CD3+, CD4+, CD8+, CD19+ and NK+ recovery post second transplant.

**Figure 3:**
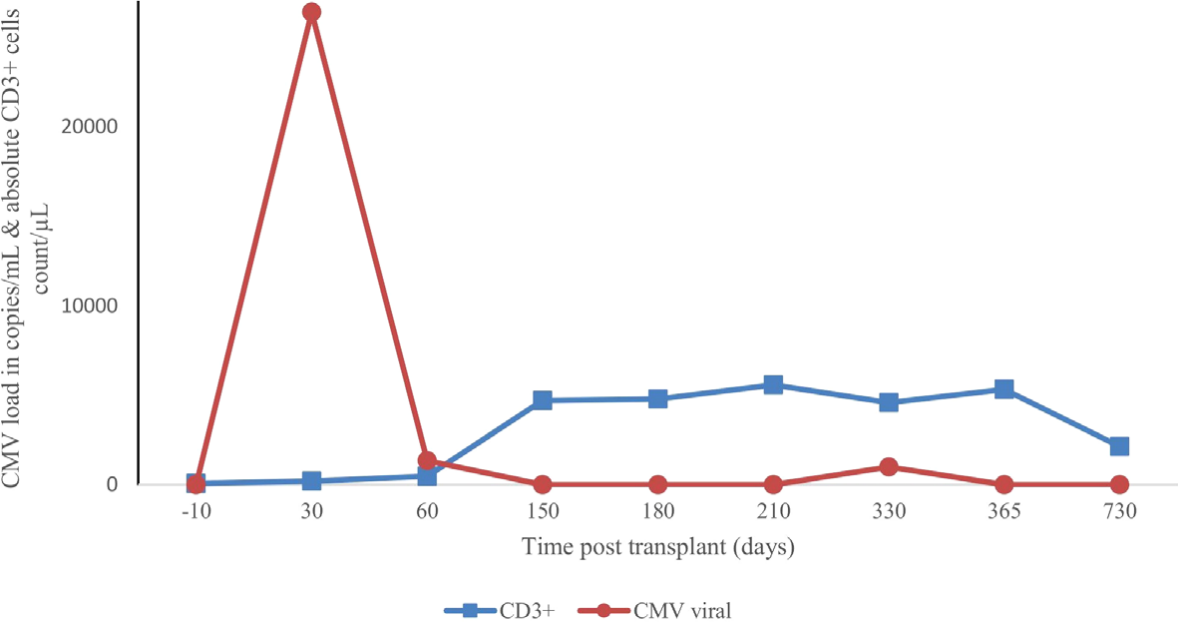
Variation in cytomegaloviral load with T-cell immune reconstitution after second transplant.

Prophylaxis against acute GVHD consisted of 50 mg/kg/day cyclophosphamide given for 2 days (days +3 and +4), tacrolimus, and MMF. During hospitalization, the patient was presented with the following complications: febrile neutropenia; hemorrhagic cystitis, which responds well to hydration, and natri 2-mercapto ethan sulfonat (mesna). Neutrophils were engrafted on day 17, and a platelet count of more than 20 × 10^9^/L was achieved on day 43 (**Figure 1**). The percentage of peripheral blood-nucleated cell chimerism was 100% on day 30. At month 6 posttransplant, immune suppression gradually tapered until it was successfully discontinued without complications. Normal levels of CD8**^+^** cells and NK cells were reached by 3 months posttransplant, whereas a normal number of CD4+ cells was achieved 7 months posttransplant. B lymphocyte (CD19^+^) counts were low or undetectable throughout the first 6 months posttransplant and increased steadily afterwards, reaching the normal range at 2 years (**Figure 2**). The patient received IVIG therapy at 500 mg/kg every 4 weeks for 12 months posttransplant. Completed hematological and immunological reconstitution was achieved 24 months after transplantation, with stable T cell, B cell, and natural killer–cell development (**Figures 1**, **2**). There was no aGVHD, and the EBV PCR (polymerase chain reaction) results remained negative after day 14. CMV was reactivated in the blood by PCR on day 1, with a maximum level of 3.52 × 10^4 cp/ml. This strain was treated with therapeutic doses of foscarnet and became undetectable on day 75 posttransplant (**Figure 3**). Ophthalmic examinations after HSCT revealed improvement in CMV retinitis. He has been in good condition for the last 36 months following HSCT, and nutritional status improved, with a weight-for-age at the 50th percentile line, height-for-age below the 3rd percentile and body mass index-for-age Z score of 1.82 SD (standard deviation). Additionally, the patient continues to have completed hematological recovery and 100% donor chimerism (**Table 1**), without evidence of GVHD. He no longer required IVIG replacement for the last 2 years.

**Table 1:** Laboratory test results post second transplant.

Index	Date									Normal range
	+30	+60	+90	+150	+210	+270	+360	+720	+1090	
WBC (10^9^/L)	4.39	3.44	8.89	11.58	13.5	11.13	12.33	6.07	9.56	6.0 - 10.5
NEU (10^9^/L)	3.52	1.10	2.29	2.45	4.62	4.71	4.24	2.25	4.85	2.5 - 6.0
LYM (10^9^/L)	0.45	1.08	3.52	6.25	7.03	5.14	6.71	3.03	3.62	1.3 - 3.5
RBC (10^12^/L)	3.0	3.03	2.94	3.52	3.63	3.91	4.41	4.39	4.55	3.92 - 4.72
PLT (10^9^/L)	75	42	76	80	163	207	212	235	276	140-440
GOT/GPT (U/L)	38.9/27.7	54/29.1	56.7/33.5	66.1/66.7	51.2/29.6	50.2/40.2	45.5/32.6	38.7/11.7	59.7/34.0	20-40
IgG (g/l)	3.99	5.81	4.22	1.5	1.74	2.58	4.11	11.01	14.11	3.45-12.36
CMV (cp/mL)	26.100	1340	(-) ve	(-) ve	(-) ve	1000	(-)ve	(-)ve	(-)ve	
EBV (cp/mL)	(-) ve	(-) ve	(-) ve	(-) ve	(-) ve	(-)ve	1000	(-)ve	(-)ve	
Chimerism (%)	100	100	100	100	100	100	100	100	100	

WBC, White blood cells; NEU, Neutrophil count; LYM, Lymphocyte count; RBC, Red blood cells; PLT, Platelet; GOT, Glutamic oxaloacetic transaminase; GPT, Glutamic pyruvic transaminase; IgG, Immunoglobulin G; CMV, Cytomegalovirus; EBV, Epstein-Barr virus.

## Discussion

Wiskott–Aldrich syndrome (WAS) is a rare, inherited immunodeficiency disorder that primarily affects males (12). Without timely diagnosis and treatment, WAS patients often face severe infections and do not survive past the age of five (1). Early intervention is critical to prevent life-threatening complications and ensuring appropriate management strategies. Overall, HSCT remains the only curative treatment for WAS. Historically, outcomes with haploidentical HSCT for WAS patients have been quite poor, with high rates of graft failure and GVHD (13). However, recent two large, multicenter studies have reported significantly outcomes of HSCT for WAS patients, including haploidentical HSCT (2, 4, 14). The Primary Immune Deficiency Treatment Consortium in the United States reported an overall survival rate of 91% at 5 years (2), whereas the European Society for Blood and Marrow Transplantation reported an 88.7% survival rate at 3 years (14). Both studies identified younger age at the time of HSCT (under 5 years old) as a critical factor, with these younger patients experiencing significantly better outcomes (2, 14). Additionally, Elfeky reported a 100% survival rate at 63 months in the United Kingdom (15). Our patient was early diagnosed WAS at 3 months old, since then he was monitored regularly by an immunologist to reduce the risk of infections and autoimmune issues prior to undergoing HSCT.

In situations where an HLA-identical donor is not available, haploidentical donors should be considered (6, 7). Primary challenge in the haploidentical HSCT setting is the risk of poor engraftment. This is largely due to the necessary depletion of T cells, which is a key component of the conditioning regimen for haploidentical HSCT essential to avoid the occurrence of GVHD (5, 6). However, extensive T-cell depletion can lead to impaired engraftment and slower immune reconstitution. The delicate balance between preventing GVHD and ensuring robust engraftment is a major challenge in haploidentical HSCT. Posttransplant cyclophosphamide not only depletes selectively alloreactive donor T cells but also preserves regulatory T cells and hematopoietic stem cells, which can facilitate engraftment (6, 8, 9). Sharma described a series of 13 patients, 10 of whom were alive and disease free (16). In China, Yue published a series of 5 patients who underwent haploidentical HSCT with a modified protocol using PTCy, busulfan, fludarabine, and antithymocyte globulin; all 5 patients were alive with 100% donor chimerism (17). Another report from Smith described two sibling patients who underwent successful T-cell replete haplo-HSCT with PTCy at 9 months and 4 years of age using their father as the donor. Myeloablative conditioning consists of rabbit anti-thymocyte globulin, busulfan, fludarabine, and melphalan (18). Other approaches include the infusion of donor natural killer cells or memory T cells to promote engraftment without triggering GVHD (2, 6, 19). Additionally, the optimization of conditioning regimens and the development of novel T-cell depletion techniques continue to be areas of active investigation (20). Currently, several trials of gene therapy for classical WAS are ongoing and are restricted to patients without a fully matched donor (21).

Inborn error of immunity (IEIs) disorders, including WAS in Vietnam face critical diagnostic challenges due to a shortage of clinical immunologists and limited access to advanced immunological and genetic testing. The country currently has only seven specialized hospitals capable of diagnosing and treating IEIs patients, which severely restricts comprehensive patient care. Since 2011, Vietnam has been systematically documenting and tracking immunodeficiency cases, and the first HSCT for IEI in Vietnam occurred in 2014 for a patient with severe combined immunodeficiency, marking important milestones in understanding and managing these complex medical conditions (7, 19). Intravenous immunoglobulin (IVIG) replacement therapy is fully covered by medical insurance for children under six years old, providing some relief for younger patients. However, late diagnosis remains a persistent problem, often resulting in delayed treatment and potential long-term health complications. The potential for HSCT is currently limited by multiple factors, including late disease detection, severe pre-existing complications, and significant organ damage. Financial issues further complicate the implementation of advanced treatment strategies for patients with IEIs. The emerging possibility of post-transplant cyclophosphamide (PTCy)-HSCT as a potential curative option highlights the urgent need for improved medical infrastructure and diagnostic capabilities. Addressing these challenges will require coordinated efforts from government health agencies, medical professionals, and international health organizations. Strategic investments in medical training, diagnostic technologies, and treatment protocols could significantly improve outcomes for patients with IEIs and WAS in Vietnam.

In our report, the conditioning regimen with antithymocyte globulin, melphalan, and fludarabine, along with posttransplant cyclophosphamide, was well tolerated, with infectious complications. Despite posttransplant cytomegalovirus (CMV) reactivation, the patient achieved 100% donor chimerism by day 30, and the CMV infection was ultimately resolved. This case adds to the growing body of evidence supporting the use of haploidentical HSCT with PTCy as a viable option for WAS patients who lack an HLA-identical donor.

## Conclusion

In conclusion, while WAS is a rare condition, it should be considered in the differential diagnosis of male patients presenting with microthrombocytopenia, eczema, and recurrent infections. Prompt diagnosis significantly enhances patient outcomes and quality of life. HSCT remains the only curative treatment, and recent advances in haploidentical HSCT with PTCy have significantly improved outcomes for WAS patients lacking a matched donor.

## Data Availability

The original contributions presented in the study are included in the article/supplementary material. Further inquiries can be directed to the corresponding author.
